# Biobased Polyethylene Hybrid Composites with Natural Fiber: Mechanical, Thermal Properties, and Micromechanics

**DOI:** 10.3390/ma13132967

**Published:** 2020-07-02

**Authors:** Patrycja Bazan, Przemysław Nosal, Barbara Kozub, Stanisław Kuciel

**Affiliations:** 1 Faculty of Materials Engineering and Physics, Institute of Materials Engineering, Tadeusz Kosciuszko Cracow University of Technology, Al. Jana Pawła II 37, 31–864 Cracow, Poland; barbara.kozub@pk.edu.pl (B.K.); stask@mech.pk.edu.pl (S.K.); 2 Department of the Strength and Fatigue of Materials and Structures, AGH University of Science and Technology, al. Mickiewicza 30, 30–059 Cracow, Poland; pnosal@agh.edu.pl

**Keywords:** natural fibers, coconut fiber, basalt fiber, wood flour, bio-polyethylene, hybrid composites, modeling

## Abstract

The work assumed the possibility of the introduction natural fibers as a hybrid reinforcement of bio-polyethylene composites. Coconut fibers, basalt fibers and wood flour were used in different combination as a hybrid merger. Mechanical tests were conducted. An increase in the mechanical properties was shown as an effect of the introduction of the fibers info the polymeric matrix. A synergic influence of hybrid reinforcement was also presented. Experimental results were compared with modeling parameters. The hydrothermal and accelerated thermal ageing effects on the mechanical behavior of composites were presented. Scanning electron microscope images were observed in order to analyze structure of examined composites.

## 1. Introduction

The rapidly growing production volume of polymer materials, caused by the increase in the scope of their applications and the use of polymer materials reinforced with particles or fibers, is associated with a significant increase in the amount of plastic waste. This provides an increasing burden on the natural environment with non-degradable materials, as well as indirectly the extraction and processing of fossil materials intended for the manufacture of plastics. Therefore, scientists as well as producers are increasingly focusing on the production of new, environmentally friendly, polymeric usable materials. These materials can be made from fossil sources as well as from renewable sources. Polymers obtained from both these sources can be completely biodegradable or resistant to this. The amount of new and ecological polymer materials that appear on the market is definitely smaller than the classic non-biodegradable polymers such as polyethylene (PE), polypropylene (PP), poly (vinyl chloride) (PVC), poly (ethylene terephthalate) (PET) or polystyrene (PS). Despite this, the global production of bioplastics, i.e., plastics produced from renewable and/or biodegradable raw materials, is important, and its growth dynamics is greater than in the case of classic polymers [1]. Biopolymers are manufactured by fermentation with the use of microorganisms by the synthesis of structural elements (monomers) from raw materials, such as lignocellulose materials, as well as organic waste and fatty acids. Natural biopolymers occur in a natural way and are the second category of biopolymers. They contain, e.g., polysaccharides (chitin, chitosan, collagen, etc.), proteins and nucleic acids [2]. Non–biodegradable plastics obtained from renewable sources, biomass, include: polypropylene, polyethylene terephthalate, polyvinyl chloride or polyethylene [3].

PE is most commonly used plastic. PE is an essential technical polymer produced from fossil raw materials. The simplicity of its production and chemical resistance make it popular in the chemical industry [4]. Polyethylene is mainly used for production of packaging such as bags, foils and plastic bottles. Its yearly output is over 60 million tons. Polyethylene is non-biodegradable. This causes ecological problems related to its usage. Recycling polyethylene is simple, although the use of a biodegradable alternative could be considered environmentally beneficial. Bio-PE is currently produced on an industrial scale from bioethanol originated from sugar cane. Other raw materials such as sugar beet, starch plants such as maize, wood, wheat and other plant waste from fermentation can be used [5].

The requirement for natural fibres has been growing in recent years. Firstly, the natural fibers are fully biodegradable, they are derived from regenerative resources, and their acquisition does not affect the climate effect. Moreover, natural fibers possess good mechanical and bio-chemical properties. The bio-chemical composition ensures that natural fibers are non–toxic and non-carcinogenic, unlike synthetic fibers, for which such properties have been noted. The best known vegetable fibers are: oil palm, wood, rice straw, bamboo, sisal, ramie, hemp, doum fruit, marc, pineapple leaf, mesta, wheat straw, cotton, flax, date palm, rice husk, curare, coconut, jovar, kenaf, rape waste, rose, banana, abaca, agave, maize and jute [6,7]. Kuciel et al. presented a positive effect of strengthening biocomposites on the basis of poly (3-hydroxybutyrate co-3-hydroxywalate) containing various natural fillers (nanocellulose, walnut shell flour, egg shell flour and tuff). The studies showed high potential of bio-composite reinforced with natural fillers [8].

Reinforcement with two or more fibers to a single matrix results in the advancement of hybrid composites with a wide variety of material properties. In the last few years, hybrid composites have been created in which more than one type, form or size of the reinforcement was introduced. Those composites have been tested in order to ensure the synergy properties of selected fillers and matrix. They provide a number of features that cannot be achieved with one type of reinforcement.

Hybridization can overcome the defects of one component by inclusion another type of fibers. The condition for the hybrid effect to occur is that the two fibers will be different due to their mechanical behavior and the interfaces between the matrix. The most useful would be to combine only two types of fiber. For example, connection of two types of short biological or cellulosic fibers with different shapes has some advantages compared to only one type of introduced fibers. Hybrid reinforcement with good fiber selection is likely to provide attractive properties and meet the present need for plastic matrix composites [9,10,11,12]. Saw and Datta developed composites with a hybrid reinforced with short bagasse and jute fiber. The results showed an increase in strength properties such as tensile and flexural strength, however, it was noted that natural fibers would better fulfill their functions after proper surface treatment [13]. Similar results were obtained by Thiruchitrambalam et al., Khanam et al., and Mwaikambo et al. in research on hybrid composites in this order: banana/kenaf as epoxy resin reinforcement, coir/silk in a polyester matrix and cotton/kapok polyester composites. In each of these works, the essence of surface treatment of the used fibers was indicated for the increase in the strength properties of the tested materials [14,15,16]. Chee et al. applied hybrid reinforcement of bamboo and kenaf fibers mat in an epoxy resin. Studies shown that the dimensional stability was strongly influenced by the fiber orientation, and the optimal combination was a 50:50 ratio of the content of bamboo and kenaf fibers [17]. Fernandes et al. examined hybrid composites based on high density polyethylene reinforced with coconut and cork. The results pointed an increase in Young’s modulus by about 30% and tensile strength by about 50% compared to unmodified material [18]. Aji et al., Athijayamani et al. and Khanam et al., worked on hybrid kenaf/pineapple reinforced composites in high density polyethylene (HDPE), sisal/roselle fiber in polyester resin and sisal / silk fiber also in polyester accordingly. They pointed to the large impact of both the shape and length of the fibers as well as the environmental conditions on the mechanical quality of hybrid composites. In each case of hybrid connection of fibers an increase in tensile and flexural properties was noted, however it was dependent on the dimensions of the reinforcement [19,20,21]. 

In past years, many studies on hybridization of artificial and vegetable lignocellulose fibers have been conducted. However, plant-fibre hybrid composites are less researched and the most frequently published reports are limited to hybrid composites containing one type of vegetable fibre and one type of artificial fibre. Examples of such reinforcements may be the use of carbon fiber in combination with flax fiber or carbon fiber with sisal fiber provided by Khanam et al. and Fiore et al. Glass fiber was also used in combination with natural fibers like banana or bamboo on a polypropylene basis in the work of Venkatasubramanian, and basalt fiber with flax, hemp or glass fiber combination were investigated by Petrucc. All presented works pointed to a great effect of hybrid reinforcement in polymer matrix composites [22,23,24,25,26].

The main gain of this work is to explore the possibility of using hybrid reinforcement made of natural fibers. This is the second part of the research conducted on the fibers of coconut, wood flour and basalt fiber introduced into the bio-polyethylene matrix. The first stage of the research involved examining the influence of natural fibers on the mechanical properties of bio-PE composites. The tests showed a positive effect of strengthening the fibers already at 6% content by weight, hence this work presents the hybridization effect of the introduced fibers. 

## 2. Materials and Methods

### 2.1. Materials

This work presents investigations on hybrid composites based on bio-polyethylene (Green PE SHC7260, Braskem, Brazil). The standard dumbbell and dogbone samples were made at Cracow University of Technology using KM 40–125 Winner Krauss Maffei (Krauss Maffei, Munich, Germany). As a reinforcement, natural fibers were used and include:

1. Basalt fiber (KV02M with a diameter of 13 µm, length about 3.2 mm, Kemenny Vek, Dubna, Russia);
2. Coconut shell fibers (Coco MLD 2 mm with a diameter of 100 µm and length about of 2 mm, Procotex S.A., Moeskoren, Belgium);
3. Wood flour (Lignocel BK 40/90 with particle size of 300–500 µm J. Rettenmaier and Söhne Company, Rosenberg Germany);
4. Coupling agent Scona TPPP 9112 FA was used (BYK, Altana, Germany).

Manufactured materials for the experiment are described in Table 1.

### 2.2. Testing Methods

Physicomechanical tests were performed to investigate strength behavior of hybrid composites based on bio-polyethylene with natural fiber. The density was calculated by the hydrostatic method using scales RADWAG WAS 22W (Radom, Poland). Mechanical tests include: static tensile test (PN-EN ISO 527-1:20100), the three-point flexural test (PN-EN ISO 178:2011) which were carried out with a MTS Criterion Model 43 universal testing machine (MTS System Corp., Eden Prairie, MN, USA), using the MTS axial extensometer. The test speed was set to 10 mm/min. A Charpy impact test (PN-EN ISO 179-1:2010) was verified on the unnotched specimens using a Zwick HIT 5.5P (Zwick Roell Group, Ulm, Germany). The values were estimated from an average of at least of 5 specimens. After the mechanical test, micrographic images were taken using a scanning electron microscope (SEM) JEOL JSN5510LV (JOEL Ltd., Tokyo, Japan). The surface chemistry of the neat polymer and composites containing natural fibers was characterized using a Fourier transform infrared (FTIR) spectrometer Thermo Scientific Nicolet iS5 FTIR spectrometer (Thermo Fisher Scientific, Waltham, Massachusetts, United States) equipped with iD7 ATR accessory (diamond monolithic crystal). Spectra were acquired at 4 cm^−1^ resolution as an average of 64 scans. The spectrum in the 4000–400 cm^−1^ range was recorded.

A water absorption calculation was carried out in accordance with ASTM D570-98. Samples were weighed periodically to saturation level using an electronic balance (RADWAG WAS 22W). At first, samples were conditioned at a temperature of 23 ± 2 °C and a humidity equal to 50 ± 5% for 24 h. The hydrothermal ageing process was undertaken in distilled water at 23 ± 2 °C. Samples were removed from the water at the following frequency: 1, 7, 14, 21 and 30 days. Before weight measurement the samples were dried by the tissue. Water absorption was calculated using the following equation:

(1) $$\%W=\frac{W_{n}-W_{0}}{W_{0}}\cdot 100$$

where *W*_0_ is the initial weight of the sample, *Wn* is the weight of the saturated sample, and %*W* is the percentage increase in weight.

Physicomechanical investigations were verified after water and thermal ageing processes. Accelerated ageing tests were proceeding according to the EN ISO 2440 in autoclave (Parr Instrument Company, Moline, IL, USA) parameters of the test include: temperature of 120 °C, humidity of 100%, and pressure of 0.3 MPa. The aging process lasted 144 h. 

To calculate the elastic modulus of hybrid composites the Voigt approximation, Reiss approximation, Hashin–Shtrikman bounds, and Halpin–Tsai model were compared with the experimental data.

## 3. Results and Discussion

### 3.1. Basic Mechanical Investigations

Strengthening and filling polymeric construction materials involve the addition of a second component to the matrix material. This effect occurs when the considered mechanical strength and rigidity of the composite increase related to the pure material, while other properties may remain unchanged or even deteriorate. Fiber reinforcement depends on the geometry of the filler, and in the case of fibers the length to diameter ratio (critical length), fiber adhesion to the matrix and other interactions such as incubation of crystalline nuclei, interatomic interactions or the ability to form chemical connections. The highest modulus value is obtained with unidirectional oriented fibers with a developed surface. Considering production of composites by means of the injection moulding process, the flow process of the material in the injection mold is quite complex, and the fiber orientation inside the sample is random, and oriented at the surface [27]. 

**Figure 1:**
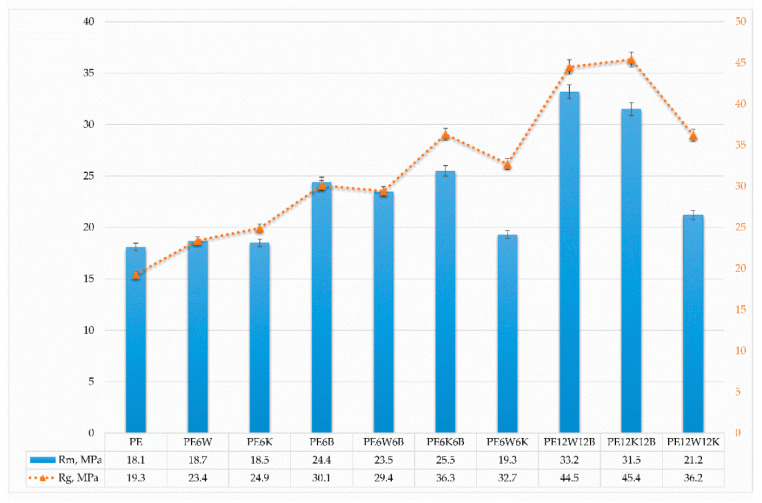
Comparison of tensile (Rm) and flexural (Rg) strength of tested materials.

**Figure 2:**
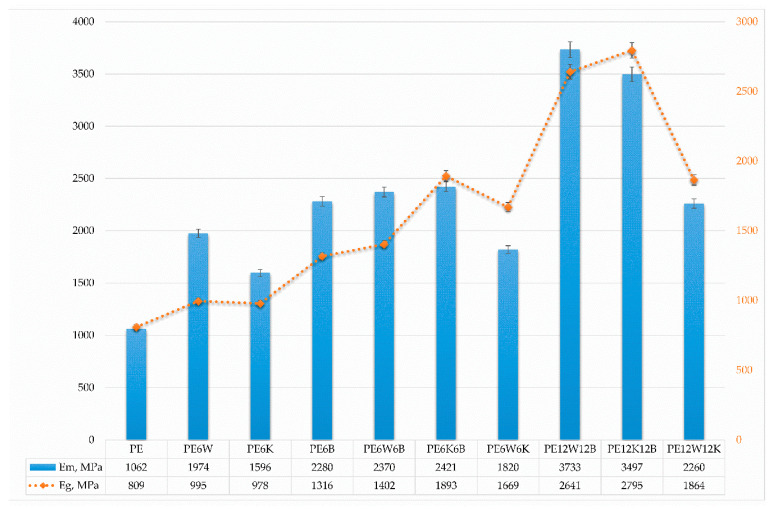
Comparison of tensile (Em) and flexural (Eg) modulus of tested materials.

**Figure 3:**
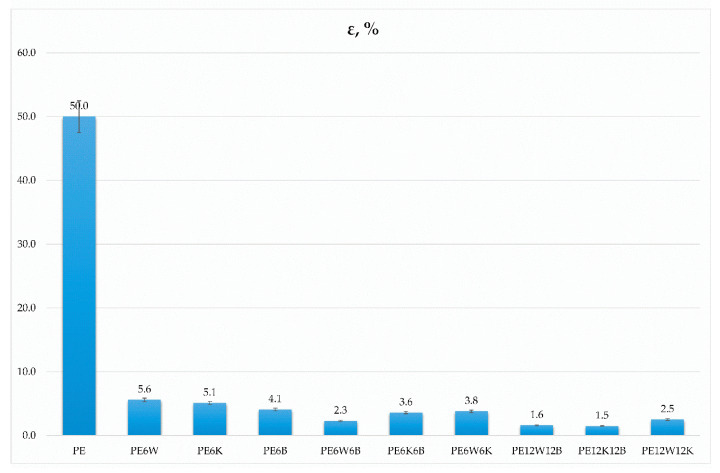
Comparison of strain at break between tested materials.

Figure 1 and Figure 2 show the values of strength and Young’s modulus depending on the direction of stress, and Figure 3 presents results of strain at break obtained from static tensile test.

Considerable differences in the properties obtained from the static tensile and flexural test result from the material loading method. In the case of the static tensile test, it is assumed that the applied stress is in accordance with the direction of the fibers. Analyzing the test results, a minor increase in strength can be explained by the fact that only a small portion of fibers with such a limited amount is oriented in the direction of force.

**Figure 4:**
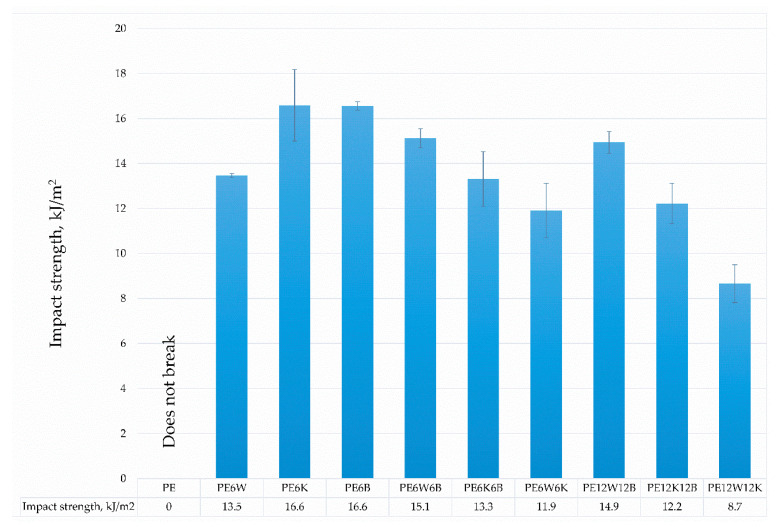
Comparison of impact strength.

Hybridization of basalt with natural fibers significantly improved mechanical properties compared with the introduction of only cellulose fibers. From the natural fibers wood flour in combination with basalt fibers had significantly higher results than basalt fibers /coconut fibers. Particularly noticeable effect was for the Young’s modulus. A twofold increment of fiber in hybrid mixtures increased reinforcement efficiency by 25–40% in most cases for the wood-basalt system, but least for the wood-coconut, which suggests a reduced adhesion of the coconut additive to the polymer material than the bond between wood flour and polyethylene, which can additionally be associated with diameter and length, and fiber surface development. The combination of wood flour and coconut fibers provided the least effect on strength properties as well as dynamic impact properties (Figure 4).

Adhesion depends on the forces at the fibre/matrix contact. Adhesion strengths are physical forces derived from the chemical constitution of the polymer matrix and filler. They can also be chemical interactions based on similarities in functional groups. The properties of the composite rely on the action of macromolecules in thin layers on the filler surface. Chain elasticity of macromolecules and changes in macromolecular conformation influence the size of adhesion, as they significantly determine the degree of contact spots between macromolecular chains and fillers [28].

The values of modulus of elasticity changed proportionally with an increase in the fibers’ contribution and were lower in a flexural test compared to the tensile modulus. The reason for such a difference is the orientation of the reinforcement in the material. In injection moulding, the fibers in the material are positioned along the material stream in the mould. During stretching, due to the characteristic arrangement of the fibers, the forces are in line with the direction of the fibre and the load is transferred from the matrix to the fibers correctly. Flexural forces, there are shear forces perpendicular to the fiber axis in the specimen, which leads to separation of the reinforcement from the matrix and introduces additional stresses [29].

### 3.2. Scanning Electron Microscopy (SEM) Investigations

**Figure 5:**
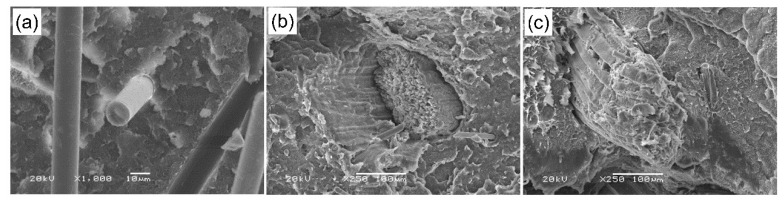
Scanning electron microscopy (SEM) images of composites with 6% wt. fiber content (**a**) polyethylene (PE) with basalt fiber, (**b**) PE with coconut fiber, (**c**) PE with wood flour.

**Figure 6:**
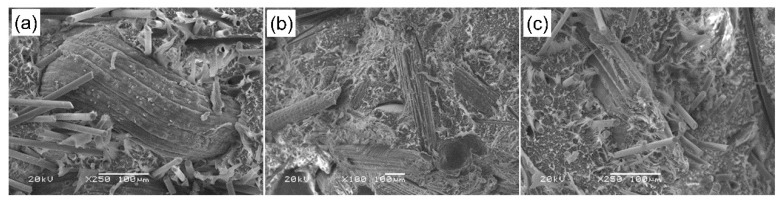
SEM images of hybrid composites with 12%wt. fiber content (**a**) PE with basalt fibers and coconut fibers, (**b**) PE with coconut fibers and wood flour, (**c**) PE with basalt fibers and wood flour.

Figure 5 and Figure 6 show SEM images of the structures of the produced composites. Figure 5a–c present composite materials contain 6% by weight of basalt fibers (a), coconut fiber (b) and (c) wood flour. Basalt fiber had a diameter of about 13 µm with a random orientation in the polyethylene matrix, and being firmly embedded in the matrix caused the fracture to be characterized by a brittle nature. Coconut fiber had a diameter in the range of 150–250 µm, the fracture surface was mixed from brittle to ductile, and the spaces between the components visible in the photo pointed low adhesion of the fiber to the polyethylene matrix. Coconut fibers and wood flour were made of single micro fibrils with a diameter of a few micrometers joined in bundles thicker and irregular for wood material, narrow and long for coconut. Microscopic images of material with the addition of wood flour revealed fairly well embedded particle sizes of diameter about 200 µm in the polymer matrix, as well as features of brittle and ductile fractures. Figure 6 shows the structure of hybrid composites. Figure 6a presents hybrid composites with basalt and coconut fiber. Significant differences in fiber diameter can be observed. In addition, the elasticity of coconut fibers and evidently high stiffness of basalt fibers are noticeable. Figure 6b,c show hybrid composites with wood flour and coconut fibers in connection with basalt fibers. The differences in the connection between additives and the matrix are also noticeable: well-embedded wood flour particles and the weak connection between the coconut fibers and polymer. Coconut fibers had a rough surface and the wood flour consisted mainly of hollow cylindrical cells, strung parallel to each other.

### 3.3. Fourier Transform Infrared (FTIR) Analysis

**Figure 7:**
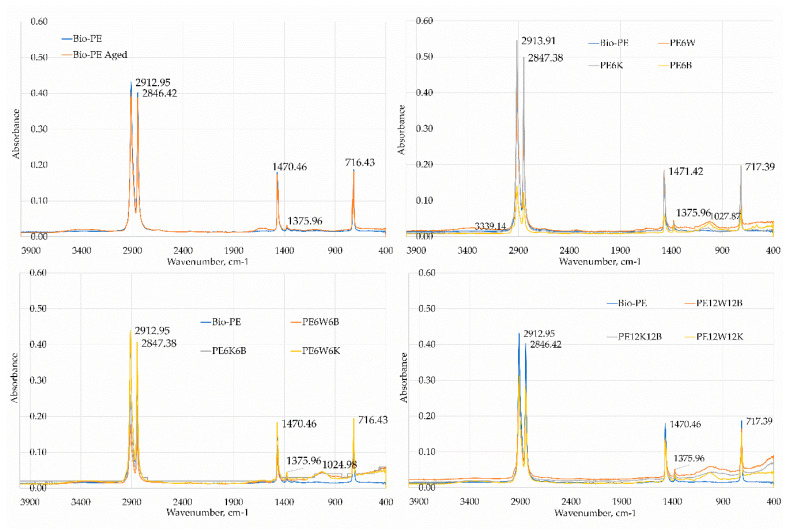
Fourier transform infrared (FTIR) spectra of tested materials.

FTIR analysis was performed with a FTIR Thermo Scientific Nicolet iS5 spectrometer equipped with an iD7 ATR (diamond monolithic crystal) accessory (Thermo Fisher Scientific, Waltham, MA USA). The tests were carried out in the 400–4000 cm^−1^ wavelength range. Figure 7 shows the FTIR spectrum for pure bio-polyethylene and composites on its matrix. The absorption band in the range of about 3000 cm ^−1^ corresponds to the vibrations of the -CH groups Polymer spectra were typical of PE with characteristic strong absorption peaks at 2912.95 cm^−1^ derived from the stretching vibrations of the C–H and 1470, 46 cm ^−1^ functional groups derived from the vibrations of the bending C–H functional groups. The presence of filler in the composite was mainly manifested by a drop in absorbance in the range 400–1500 cm^−1^ and 2600–3100. A comparison of the FTIR spectra of the raw polymer and polymer composites showed that there were no shifts at the position of the absorption peaks, and suggested that the potential interaction of the fiber with the polymer matrix was elatively low. 

### 3.4. Temperature Influence and Ageing Process

Construction materials are used in various environments, including changing temperatures, humidity and mechanical loads. Since the softening process in semicrystalline plastics starts smoothly in amorphous areas and then the crystalline areas melt. The establishment of thermal boundaries of application is a very hard condition during designing structural elements.

**Figure 8:**
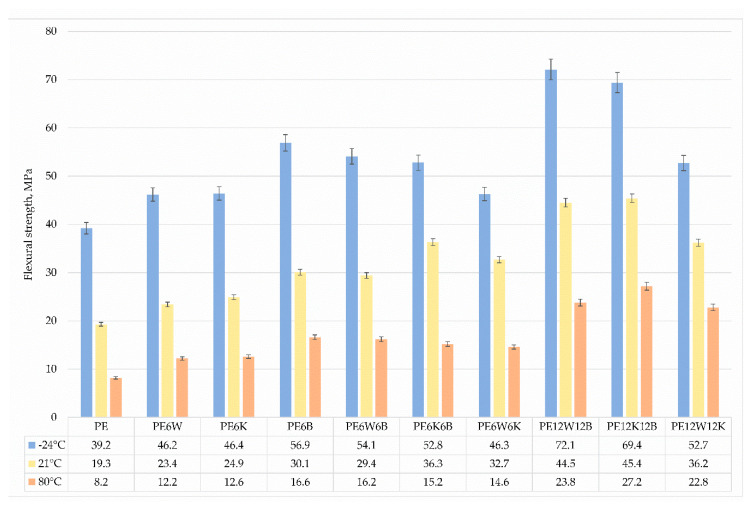
Comparison of flexural strength at three different temperatures.

**Figure 9:**
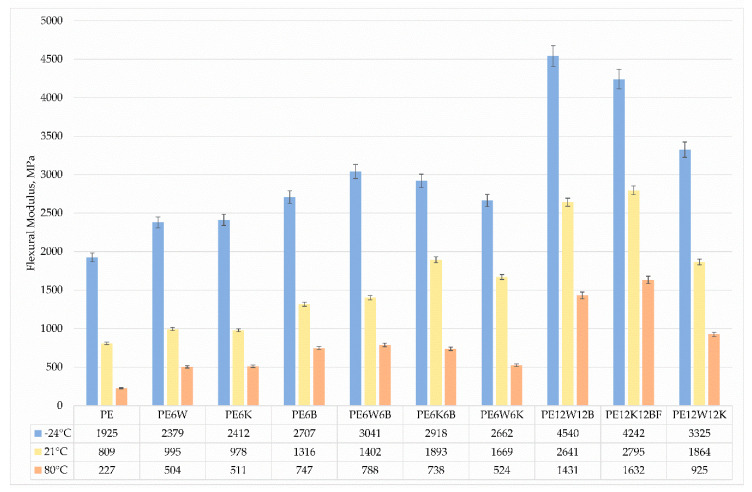
Comparison of flexural modulus at three different temperatures.

The static flexural test was carried out at 3 different temperatures in the range of extreme working temperatures to show the influence of temperature on changes of strength abilities. Results are presented in Figure 8 and Figure 9. As the temperature increased, the strength and the flexural modulus decreased. Maximum strength properties were achieved for materials with basalt fiber. It should also be noted that composites with a combination of coconut and basalt fibers achieved higher strength properties at ambient temperature and at 80 °C, but at negative temperatures the addition of wood flour provided higher strength properties. Most likely, this is related to particle geometry. 

Analyzing the flexural modulus, higher parameters are obtained when the reinforcement is in the form of particles, and works by blocking the movements of polymer segments. The neat matrix also provides increased rigidity. At low temperatures, the particles are not very mobile and cannot respond to vibrations and remain rigid. It is said that Brown micromovements have been frozen, which means that no changes of macromolecule segments is possible, especially around C–C bonds, and that the looping of these molecules act as crosslinking points. At higher temperatures, sections of the macromolecules become more mobile and can easily respond to loads, loops still persist, but may react in some cases by slipping and the macromolecules slip relative to each other, reducing strength and stiffness, but also causing an increase in deformability [30].

The materials were also subjected to a water absorption test. This study is particularly important for biopolymers that are used in applications as structural materials under thermally or mechanically loaded conditions. Water molecules penetrate deeply into the material mass and settle mainly in less-packed amorphous areas due to the formation of hydrogen bridges. The lower the crystalline phase, the greater the moisture absorption capacity. Polyethylene, in principle, is a material that does not absorb water, however the introduction of fibers or particles, especially cellulosic, causes water absorption inside the material, because the fibers act as water-carrying capillaries, and in addition, natural particles and fibers strongly absorb water by swelling and introduce stresses inside the material.

**Figure 10:**
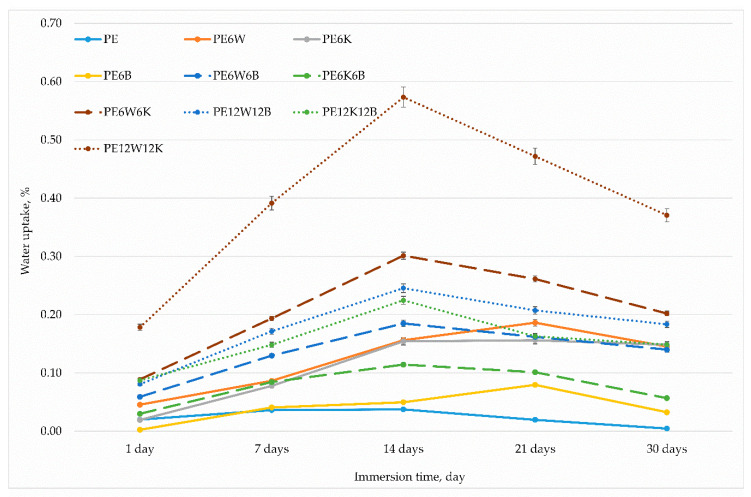
Water absorption after temporary immersion of samples in water.

Figure 10 shows water uptake for the tested materials. A huge increase in water absorption for materials that contained a high proportion of coconut-wood fillers can be noticed. Materials with the addition of basalt fiber had the lowest water content, which confirms the high moisture absorption capacity of cellulose fibers and the lack of this capacity for basalt fibers. A high removal degree of natural fibers from the matrix after 14 days of immersion of the samples in water was also noticeable. Poor adhesion between natural fibers and stresses caused by water ingress caused the the fibers to be pulled out and removed them from the material which is seen as a decrease in water uptake measurement. The results from the strength tests (presented in Table 2) showed a clear increase of Young’s modulus of several percent after soaking the samples in water, which is the effect of increasing the volume of fibers and the formation of stresses between the wetted fiber and the polymer matrix. A smaller but noticeable 5–15% increase in strength was also visible, except for materials contained only cellulose additives.

Highly important changes in the case of plastics and composites on their matrix are changes caused by ageing processes, because they largely define the suitability of the material to work in a given environment, and the lifetime of such a material. Aging of polymers is the decomposition of polymers under the influence of the sum of physical and chemical factors that affect them during usage and storage. In linear polymeric materials, as a result of degradation, the chain of macromolecules is shortened and consequently the molar mass decreases. In the polymers with a more complex chain structure, apart from the processes of breakage of the main polymer chain, there are also side group fracture reactions. As a result of physical factors such as temperature or moisture, a destructive change in the chemical and physical structure of the polymers occurs, resulting in changes in properties [32]. 

Table 2 aslo presents the results from a static tensile test carried out after accelerated thermal ageing. The mechanical properties of hybrid materials were greatly reduced when the materials were exposed to both moisture and elevated temperature. Water entering the material caused weakening of the connection between the components of the composite, and the increase in temperature accelerated the diffusion speed the degradation effect of these materials, especially for composites with cellulose fibers, which are particularly exposed to this type of process.

### 3.5. Modeling of Effective Properties

An important issue related to creating a new material is the ability to predict its properties at every stage of design. In the case of materials subjected to mechanical loads, key parameters are the magnitude of Young’s modulus and ultimate tensile strength. When considering composite materials, it should be kept in mind that their mechanical properties at the macro scale are the result of a certain combination of properties of each component [33]. The Voigt [34] and Reuss [35] models were the first approximations trying to describe this phenomenon. Among others, Voigt described the relationship of the average Young’s modulus in a function of the volume fraction of the strengthening phase. It was a simple linear model that well described brittle phase-reinforced composites, but in the case where the reinforcement exhibited more plastic behavior, this approximation gave large discrepancies. This effect has been eliminated in part by using a different approach when describing the relationship of properties, namely the average harmonic. This model is non-linear and is known as Reuss approximation. It should be noted that both cases described a two-phase composite. In order to describe a multi-phase composite, some formulas should be modified:

(2) $${\bar{E}}^{V}=\overset{k}{\underset{r=0}{\sum }}\phi^{r}E^{r}$$

(3) $${\bar{E}}^{R}={\left[\overset{k}{\underset{r=0}{\sum }}\phi^{r}{\left(E^{r}\right)}^{-1}\right]}^{-1}$$

Currently, both the Voigt (V) model and the Reuss (R) model are rarely used to estimate the properties of the composite, but rather constitute certain boundaries of the space in which the estimated parameters are located. This half-space is filled with numerous micromechanical models that have been derived from the continuum mechanics [36,37,38,39]. One of these models is the Hashin–Shtrikmann model, which, like the approximations previously described, sets the upper and lower limits in the described mechanical properties [36]:

(4) $$\kappa_{m}+\frac{\phi_{f}}{\frac{1}{\kappa_{f}-\kappa_{m}}+\frac{\left(1-\phi_{f}\right)}{\kappa_{m}+\mu_{m}}}\leq \kappa^{*}\leq \kappa_{f}+\frac{1-\phi_{f}}{\frac{1}{\kappa_{m}-\kappa_{f}}+\frac{\phi_{f}}{\kappa_{f}+\mu_{f}}}$$

(5) $$\mu_{m}+\frac{\phi_{f}}{\frac{1}{\mu_{f}-\mu_{m}}+\frac{\left(1-\phi_{f}\right)\left(\kappa_{m}+2\mu_{m}\right)}{2\mu_{m}\left(\kappa_{m}+\mu_{m}\right)}}\leq \mu^{*}\leq \mu_{f}+\frac{1-\phi_{f}}{\frac{1}{\mu_{m}-\mu_{f}}+\frac{\phi_{f}\left(\kappa_{f}+2\mu_{f}\right)}{2\mu_{f}\left(\kappa_{f}+\mu_{f}\right)}}$$

where μ means shear modulus, and κ plane strain bulk modulus, $\phi_{f}$—volume fraction, m, f—mean matrix and fibers, respectively.

Another model that is widely used to homogenize the elastic parameters of the composite is the Halpin-Tsai model [39]. This is the model most often used to describe composites reinforced with short fibers; however, it was developed assuming the reinforcement with long fibers. In the event that the short fibers are arbitrarily oriented, it uses the formula:

(6) $$E_{c}=E_{m}\left[\frac{3}{8}\left(\frac{1+\zeta \eta_{L}\phi_{f}}{1-\eta_{L}\phi_{f}}\right)+\frac{5}{8}\left(\frac{1+2\eta_{T}\phi_{f}}{1-\eta_{T}\phi_{f}}\right)\right]$$

where directional parameters $\eta_{L}$ and $\eta_{T}$ are determined by formulas:

(7) $$\eta_{L}=\frac{E_{f}/E_{m}-1}{E_{f}/E_{m}+\zeta }$$

(8) $$\eta_{T}=\frac{E_{f}/E_{m}-1}{E_{f}/E_{m}+2}$$

(9) ζ=2l/d

The parameter ζ describes the geometrical relationship between the fiber length and its diameter: In the presented work, the Young’s modulus of individual hybrid composites obtained in an experimental way was compared with the estimates described by the Voigt, Reuss and Halpin- The parameter ζ describes the geometrical relationship between the fiber length and its diameter: In the presented work, the Young’s modulus of individual hybrid composites obtained in an experimental way was compared with the estimates described by the Voigt, Reuss and Halpin–Tsai (H–T) models. At the same time, a two-stage homogenization method was used for the Halpin–Tsai model. In this method, the strengthening phases were divided into individual stages of averaging properties. Thus, in the first stage, the H–T model used one of the components of the strengthening phase to estimate the initial averaged Young’s modulus. In the second stage, this module was treated as a parameter of the average matrix in which the second of the strengthening phases was placed. Then, after re–approximation of Halpin–Tsai, the resultant values of the Young’s modulus of the hybrid composite were obtained.

**Figure 11:**
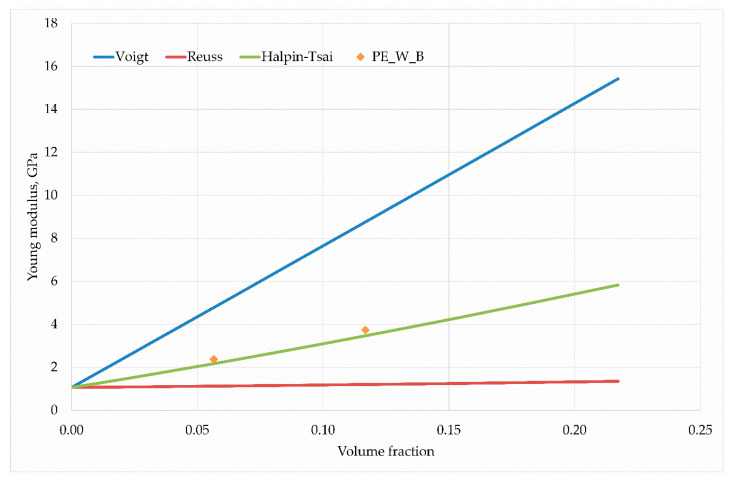
Young’s modulus dependence on the wood flour and basalt fiber content in the PE matrix.

**Figure 12:**
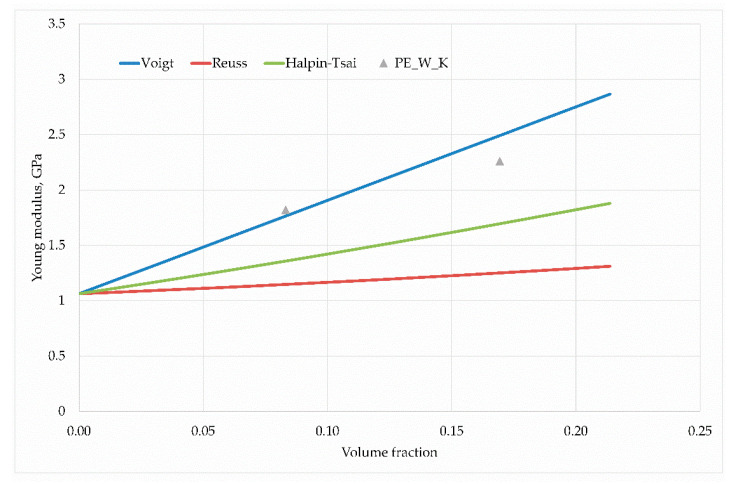
Young’s modulus dependence on the wood flour and coco fiber content in the PE matrix.

**Figure 13:**
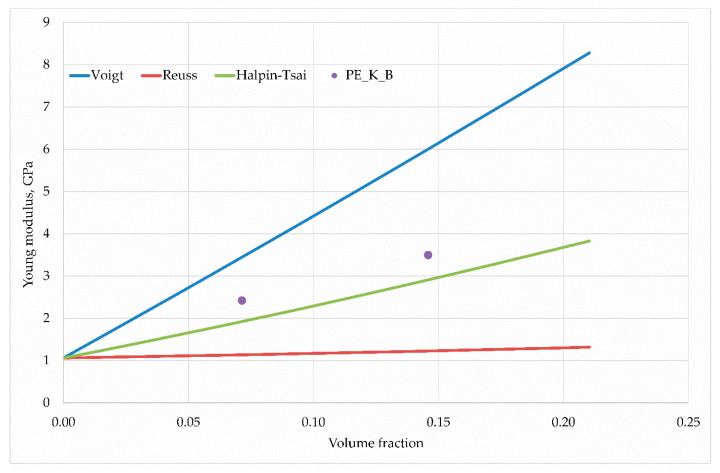
Young’s modulus dependence on the coconut fiber and basalt fiber content in the PE matrix.

When calculating the volume fraction of individual components in the composite, the following formula was used:

(10) $$\phi_{f}=\frac{1}{1+\frac{\rho_{f}}{\rho_{m}}\left(\frac{1}{\mathrm{wt}}-1\right)}$$

in which $\rho_{f}$, $\rho_{m}$, wt represent the density of the reinforcing phase, matrix density and the weight proportion of the reinforcing phase, respectively. For the hybrid composite case, the volume fractions of the reinforcing phases were first estimated and then the matrix volume fraction was calculated. Figure 11, Figure 12 and Figure 13 present the results of calculations carried out for individual hybrid composites.

In each case, the H–T model describes the elastic properties sought for well. The trend associated with the increasing non-volume share of reinforcing phases is correct; however, only in the case of hybrid composites based on polyethylene with wood flour and basalt fibres(PE–W–B) there is a small estimation error quantitatively.

The case of composite reinforced with fibers of coconut and wood flour is worth noting and thinking about. In this case, for a smaller volume fraction, the obtained Young’s modulus value exceeded the theoretical value of the Voigt model, which is generally accepted as the upper limit of the searched space of averaged values. This result could be related to the fact that in some cases the Poisson effect can lead to estimations which are beyond the Voigt approximation [40]. The discrepancies in the experimental and model results are most likely due to the geometry of the wood flour particles, because in this issue the diameter coincides with the particle length, which reduces the value of the Young’s modulus. A larger ratio of fiber length to its diameter is responsible for higher modulus values. Nevertheless, the results of the H–T model coincide qualitatively with the experiment, only differing quantitatively, which can be caused by the ratio of fiber length to diameter.

## 4. Conclusions

The presented studies on the natural fibers used as a filler in a hybrid reinforced bio-polyethylene composite showed higher rigidity and strength properties. The best results were obtained for basalt fiber-reinforced material and pointed to an increase in strength by about 30% and more than a twofold increase in stiffness. However, the purpose of this work was to assess the suitability of natural fibers in a hybrid combination to obtain lightweight structural composites. Hybridization of cellulose fibers with basalt fibers significantly improved the properties compared to the introduction of cellulose fibers alone. From natural fibers, wood flour in this combination had much better results than coconut fibers, an increase in strength by 50% and stiffness by about 65% compared to the basalt–coconut combination. The effect was particularly visible in the case of Young’s modulus. Research after accelerated thermal and water aging showed the scale of changes occurred in composite materials based on a polyethylene matrix. Accelerated aging caused a decrease in strength properties compared to materials not subject to this process. The research is interesting because it shows the potential for using renewable sources to produce lightweight composite materials with good strength properties. There has been little research on the subject of hybrid composites based on bio-polyethylene. This work allowed us to assess the use of natural fibers in experimental research and the usefulness of mathematical models to predict gain effects. Further research by researchers will focus on a deeper understanding of the effects of material aging to minimize the impact of the external environment on the properties of these materials.

## Figures and Tables

**Table 1:** Description of tested materials.

Index	Description—the Ratio of Ingredients Included wt%	Density g/cm^3^
PE	100 wt% bio-polyethylene	0.95
PE6W	92 wt.% of Bio-PE + 6 wt.% wood flour + 2 wt.% Scona TPPP 9112 FA	0.98
PE6K	92 wt.% of Bio-PE + 6 wt.% coconut shell fibers + 2 wt.% Scona TPPP 9112 FA	0.97
PE6B	92 wt.% of Bio-PE + 6 wt.% basalt fibers + 2 wt.% Scona TPPP 9112 FA	0.99
PE6W6B	86 wt.% of Bio-PE + 6 wt.% wood flour + 6 wt.% basalt fibers+ 2 wt.% Scona TPPP 9112 FA	1.01
PE6K6B	86 wt.% of Bio-PE + 6 wt.% coconut shell fibers + 6 wt.% basalt fibers+ 2 wt.% Scona TPPP 9112 FA	1.01
PE6W6K	86 wt.% of Bio-PE + 6 wt.% wood flour + 6 wt.% coconut shell fibers + 2 wt.% Scona TPPP 9112 FA	0.98
PE12W12B	74 wt.% of Bio-PE + 12 wt.% wood flour + 12 wt.% basalt fibers+ 2 wt.% Scona TPPP 9112 FA	1.06
PE12K12B	74 wt.% of Bio-PE + 12 wt.% coconut shell fibers + 12 wt.% basalt fibers+ 2 wt.% Scona TPPP 9112 FA	1.05
PE12W12K	74 wt.% of Bio-PE + 12 wt.% wood flour + 12 wt.% coconut shell fibers + 2 wt.% Scona TPPP 9112 FA	1.02

**Table 2:** Compassion of strength properties after hydrothermal and accelerated ageing process.

Index	Properties After Hydrothermal Ageing			Properties After Accelerated Ageing Process		
	Tensile Strength, MPa	Tensile Modulus, MPa	Strain at Break, %	Tensile Strength, MPa	Tensile Modulus, MPa	Strain at Break, %
Bio-PE *	21.3 ± 0.1	1210 ± 27	>100	18.7 ± 0.9	1315 ± 26	4.0 ± 0.6
PE6W *	21.4 ± 0.1	1818 ± 106	5.3 ± 0.3	15.2 ± 0.1	1644 ± 21	2.3 ± 0.1
PE6K *	21.0 ± 0.1	1727 ± 203	5.1 ± 0.7	13.2 ± 0.1	1621 ± 86	1.8 ± 0.1
PE6B *	27.8 ± 1.2	2134 ± 97	4.0 ± 0.2	20.6 ± 1.2	2745 ± 162	1.8 ± 0.3
PE6W6BF	23.8 ± 0.6	2746 ± 57	2.1 ± 0.1	20.2 ± 2.3	2463 ± 23	2.7 ± 0.3
PE6K6BF	22.1 ± 0.8	2267 ± 241	2.4 ± 0.1	18.0 ± 1.1	2177 ± 21	2.7 ± 0.2
PE6W6K	19.2 ± 0.3	1874 ± 39	3.7 ± 0.1	15.1 ± 1.3	1833 ± 32	2.3 ± 0.4
PE12W12BF	28.2 ± 0.5	3599 ± 120	2.0 ± 0.2	22.7 ± 0.7	3871 ± 43	1.2 ± 0.1
PE12K12BF	26.6 ± 2.1	3238 ± 335	1.7 ± 0.01	20.4 ± 1.5	3111 ± 15	1.3 ± 0.1
PE12W12K	19.8 ± 0.4	2359 ± 228	2.8 ± 0.3	13.2 ± 0.9	2098 ± 47	1.4 ± 0.2

* Results obtained from first stage of research on natural fibers and bio-polyethylene detailed presented in [31].
